# *Monilochaetes pteridophytophila* (Australiascaceae, Glomerellales), a new fungus from tree fern

**DOI:** 10.3897/BDJ.9.e67248

**Published:** 2021-07-30

**Authors:** Jingyi Zhang, Rungtiwa Phookamsak, Ausana Mapook, Yongzhong Lu, Menglan Lv

**Affiliations:** 1 School of Food and Pharmaceutical Engineering, Guizhou Institute of Technology, Guiyang, China School of Food and Pharmaceutical Engineering, Guizhou Institute of Technology Guiyang China; 2 Center of Excellence in Fungal Research, Mae Fah Luang University, Chiang Rai, Thailand Center of Excellence in Fungal Research, Mae Fah Luang University Chiang Rai Thailand; 3 School of Science, Mae Fah Luang University, Chaing Rai, Thailand School of Science, Mae Fah Luang University Chaing Rai Thailand; 4 East and Central Asia Regional Office, World Agroforestry Centre (ICRAF), Kunming, China East and Central Asia Regional Office, World Agroforestry Centre (ICRAF) Kunming China; 5 Centre for Mountain Futures (CMF), Kunming Institute of Botany, Kunming, China Centre for Mountain Futures (CMF), Kunming Institute of Botany Kunming China; 6 Research Center of Microbial Diversity and Sustainable Utilization, Faculty of Sciences, Chiang Mai University, Chiang Mai, Thailand Research Center of Microbial Diversity and Sustainable Utilization, Faculty of Sciences, Chiang Mai University Chiang Mai Thailand; 7 Honghe Center for Mountain Futures, Kunming Institute of Botany, Chinese Academy of Sciences, Honghe, China Honghe Center for Mountain Futures, Kunming Institute of Botany, Chinese Academy of Sciences Honghe China

**Keywords:** one new taxon, Hyphomycetes, Pteridophytes, Sordariomycetes, taxonomy

## Abstract

**Background:**

During taxonomic and phylogenetic studies of fungi on pteridophytes in Thailand, *Monilochaetes pteridophytophila* sp. nov. was collected from the frond stalks of a tree fern (*Alsophila costularis*, Cyatheaceae). The new species is introduced, based on evidence from morphology and phylogenetic analyses of a concatenated dataset of LSU, ITS, SSU and RPB2 sequences.

**New information:**

*Monilochaetes pteridophytophila* differs from extant species of *Monilochaetes* in having darker conidiophores with fewer septae (1–4-septate). *Monilochaetes pteridophytophila* forms a distinct clade, basal from other species of *Monilochaetes* in Australiascaceae. A detailed description and illustrations of the new species are provided. We also provided a synopsis of accepted species of *Monilochaetes*.

## Introduction

Studies on the diversity of fungi on pteridophytes have revealed many new taxa during the last decade (Mehltreter 2010, Braun et al. 2013, Kirschner and Liu 2014, Guatimosim et al. 2016, Kirschner et al. 2019). An estimated 670 species of fern occur in Thailand (Lindsay and Middleton 2009), making it a suitable area for studying the fungi associated with ferns. However, the study of fungi on ferns is in its infancy (Razikin et al. 2014, Kirschner et al. 2019). Cyatheaceae, a family of scaly tree ferns in Cyatheales, is widely distributed in tropical and subtropical areas (Lehnert 2011, Korall and Pryer 2014). Species of Cyatheaceae diverged ca. 150 (146–168) million years ago during the Late Jurassic period (Korall and Pryer 2014). Many taxa in this family are threatened species, including *Cyathea brunoniana*, *C. gigantea* and *C. henryi* (Balkrishna et al. 2020, Coritico and Amoroso 2020).

*Monilochaetes* Halst. ex Harter was introduced by Harter (1916) to accommodate a pathogenic fungus, *M. infuscans* Harter, that caused scurf disease of the sweet potato. *Monilochaetes infuscans* was first reported by Halsted (1890), but the species is considered invalid due to the lack of morphological description and illustrations. Réblová et al. (2011a) established the family Australiascaceae Réblová & W. Gams to accommodate *Australiasca* Sivan. & Alcorn (as a sexual morph) and *Monilochaetes* (as an asexual morph). Sivanesan and Alcorn (2002) introduced *Australiasca* with *A. queenslandica* Sivan. & Alcorn as the type species, which was linked to *Dischloridium camelliae* Alcorn & Sivan as an asexual morph. Réblová et al. (2011a) treated *Dischloridium* B. Sutton as the generic synonym of *Monilochaetes*, based on phylogenetic analysis of ITS and LSU sequences. Following the “One Fungus One Name” (1F1N) principle, *Australiasca* was synonymised under *Monilochaetes*, the latter being older (Réblová et al. 2016, Hyde et al. 2020a). Hyde et al. (2020a) and Wijayawardene et al. (2020) accepted Australiascaceae in Glomerellales with a single genus *Monilochaetes.* Index Fungorum (2021) lists nine species in *Monilochaetes*. These are *M. basicurvata* (Matsush.) Réblová & Seifert, *M. camelliae* (Alcorn & Sivan.) Réblová, W. Gams & Seifert, *M. dimorphospora* Réblová & W. Gams, *M. guadalcanalensis* (Matsush.) I.H. Rong & W. Gams, *M. infuscans*, *M. laeënsis* (Matsush.) Réblová, W. Gams & Seifert, *M. melastomae* Crous, *M. nothapodytis* S.X. Zhou, J.C. Kang & K.D. Hyde and *M. regenerans* (Bhat & W.B. Kendr.) Réblová & Seifert. Of those, seven species have molecular data in NCBI GenBank (Sivanesan and Alcorn 2002, Réblová et al. 2011a, Réblová et al. 2011b, Zhou et al. 2017, Crous et al. 2018).

The sexual morph of *Monilochaetes* is characterised by superficial, dark brown, obpyriform perithecia with or without setae, with periphysate ostioles; hyaline, branching, septate paraphyses; 8-spored, unitunicate, cylindrical-clavate, short-pedicellate asci; and hyaline, ellipsoidal to ovoid, 0–3-septate ascospores (Sivanesan and Alcorn 2002, Réblová et al. 2011a). The asexual morph of *Monilochaetes* is characterised by solitary, erect, sometimes curved or geniculate, septate, pale brown to dark brown conidiophores; phialidic, terminal, hyaline to pale brown, ampulliform to cylindrical conidiogenous cells with a shallow collarette; and hyaline, aseptate or rarely septate, oval conidia (Harter 1916, Bhat and Kendrick 1993, Réblová et al. 2011a, Réblová et al. 2011b, Zhou et al. 2017, Crous et al. 2018).

In this study, a new species of *Monilochaetes*, *M. pteridophytophila*, is described, illustrated and compared with closely-related taxa. Morphological study and multilocus phylogenetic analyses confirm the identity of the new species and confirm its placement in *Monilochaetes*.

## Materials and methods

**Sample collection, isolation and conservation**

Frond stalks of *Alsophila costularis* (tree fern) were collected in a disturbed forest near the roadside in Tak Province, Thailand. Specimens were packed into a plastic bag for transportation to the laboratory and the associated metadata were noted (date, locality and host). Fungal colonies on the host surface were observed and examined using a stereomicroscope (Leica EZ4, Leica Microsystems AG, Singapore). Micro-morphological characters were documented with a Nikon DS-Ri2 digital camera fitted to a Nikon ECLIPSE Ni compound microscope (Nikon, Japan). Measurements of morphological structures (conidiophores, conidiogenous cells and conidia) were made with the Tarosoft (R) Image Frame Work. Figures were processed and combined with Adobe Illustrator CS6 (Adobe Systems, USA).

Single spore isolation was carried out to obtain a pure culture, following the method described by Dai et al. (2017). Germinated conidia were aseptically transferred to potato dextrose agar (PDA) plates and incubated at 25°C. Cultures were grown for 2 weeks and culture characteristics, such as size, shape, colour and texture, were recorded. The holotype specimen and ex-type living culture are deposited in the Herbarium of Mae Fah Luang University (MFLU) and Mae Fah Luang University Culture Collection (MFLUCC), Chiang Rai, Thailand, respectively. An isotype specimen is deposited at the Herbarium of Guizhou Academy of Agricultural Sciences (GZAAS), Guiyang, China.

**DNA extraction, PCR amplification and sequencing**

Fresh fungal mycelium grown on PDA at 25°C for 2 weeks was used to extract DNA. Genomic DNA was extracted by using the Biospin Fungus Genomic DNA Extraction Kit (BioFlux, China), following the manufacturer’s instructions. We amplified the internal transcribed spacer (ITS) region, the small and large subunits of the ribosomal RNA gene (SSU, LSU) and the second largest subunit of RNA polymerase II (RPB2). Primer pairs and PCR thermal cycle conditions are listed in Table 1. The quality of PCR products was checked on 1% agarose gel electrophoresis stained with ethidium bromide. Successful PCR products were sent to Sangon Biotech (Shanghai, China) for purification and sequencing. Forward and reverse sequence reads were assembled using SeqMan v. 7.0.0 (DNASTAR, Madison, WI). Consensus sequences were submitted to NCBI GenBank (Table 2).

**DNA sequence alignments and phylogenetic analysis**

Closely-related taxa were selected for phylogenetic analyses, based on BLASTn searches in NCBI GenBank (https://blast.ncbi.nlm.nih.gov/Blast.cgi), as well as recent publications (Réblová et al. 2011a, Hongsanan et al. 2017, Zhou et al. 2017, Crous et al. 2018, Dissanayake et al. 2020, Table 2). Sequences of each locus were aligned using the online multiple alignment programme MAFFT version 7 (https://mafft.cbrc.jp/alignment/server/, Katoh et al. 2019) and then manually adjusted in BioEdit 7.1.3.0 (Hall 1999). Phylogenetic relationships were inferred, based on a combined LSU–ITS–SSU–RPB2 dataset. Sequences of each locus were combined to form a concatenated super matrix using SequenceMatrix 1.7.8 and analysed with Maximum Likelihood (ML) and Bayesian Inference (BI) criteria.

Maximum Likelihood (ML) analysis was performed using IQ-TREE (Nguyen et al. 2015, Chernomor et al. 2016) under partitioned models. The optimal nucleotide substitution model for each locus was selected under the corrected Akaike Information Criterion (AICc) using jModelTest2 (Darriba et al. 2012) on XSEDE via the CIPRES Science Gateway 3.3 (https://www.phylo.org/portal2/home.action, Miller et al. 2010). The TIM3+I+G model (-lnL = 3601.7319) was selected for LSU, GTR+I+G (-lnL = 4351.9427) for ITS, TIM1+G (-lnL = 2071.9778) for SSU and TIM2+I+G (-lnL = 7734.2580) for RPB2. A non-parametric bootstrap (BS) analysis was implemented with 1000 replicates (Hoang et al. 2018).

The aligned fasta file was converted to nexus file format for BI analyses using AliView. BI analyses were performed in CIPRES (Miller et al. 2010) with MrBayes on XSEDE 3.2.7a (Ronquist et al. 2012). The best-fit evolutionary model for BI analysis was determined using MrModeltest v.2 (Nylander 2004). For the LSU, ITS and RPB2 datasets, GTR+I+G was selected, whereas GTR+G was selected for SSU. Bayesian posterior probabilities (PP) (Rannala and Yang 1996) were evaluated, based on Markov Chain Monte Carlo (MCMC) sampling. Four simultaneous Markov chains were run for 10,000,000 generations and trees were sampled every 1,000th generation (yielding 10,000 total trees). The first 2,500 trees, which represented the burn-in phase of the analysis, were discarded. The remaining 7,500 trees were used to calculate PP in the majority rule consensus tree.

Phylogenetic trees were visualised using FigTree v. 1.4.0 (Rambaut and Drummond 2008) and edited using Microsoft Office PowerPoint 2010 and Adobe Illustrator CS6 (Adobe Systems, USA). The final alignments and trees were deposited in TreeBASE (http://www.treebase.org/, accession number: 27987).

## Taxon treatments

### Monilochaetes pteridophytophila

J.Y. Zhang, K.D. Hyde & Y.Z. Lu sp. nov.

8F826744-3C0A-56D1-9E08-B4AC11C9E31A

IF558296

Facesoffungi number: FoF 09708

#### Materials

- **Type status:** Holotype. **Occurrence:** recordedBy: Jing Yi Zhang; **Taxon:** scientificName: *Monilochaetes pteridophytophila*; phylum: Ascomycota; class: Sordariomycetes; order: Glomerellales; family: Australiascaceae; **Location:** locationRemarks: THAILAND, Tak Province, Umphang District, Mo Kro Subdistrict, 16°12'11"N, 98°52'5"E, 21 August 2019; **Event:** habitat: Terrestrial; fieldNotes: on dead frond stalks of *Alsophila costularis* Baker (Cyatheaceae) in a disturbed forest nearby the roadside; **Record Level:** collectionID: MFLU 21–0023; collectionCode: Y26
- **Type status:** Isotype. **Record Level:** collectionID: GZAAS 21-0015

#### Description

**Figure 1.**
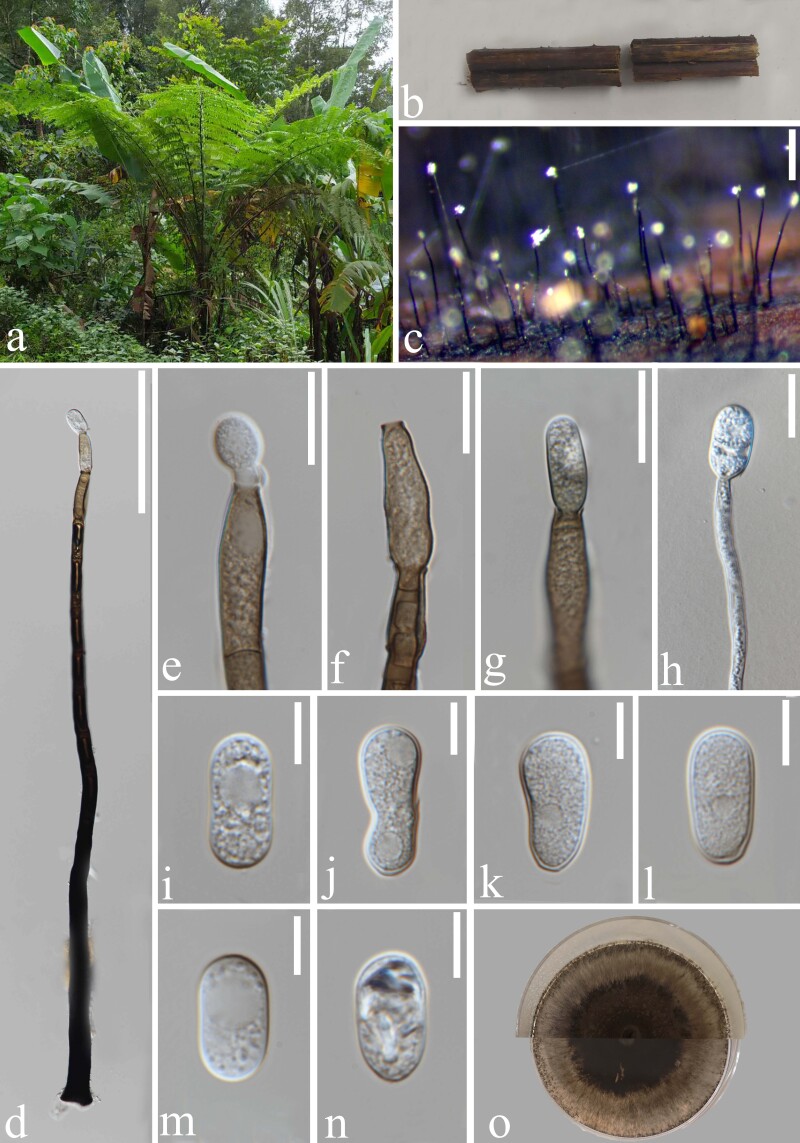
*Monilochaetes pteridophytophila* (MFLU 21-0023, holotype). **a.** The host tree fern (*Alsophila costularis*) in the field; **b.** Dead frond stalks of tree fern; **c.** Colony on dead frond stalk of tree fern; **d.** Conidiophore; **e**–**g.** Conidiogenous cells with attached conidia; **h.** Germinating conidium; **i**–**n.** Conidia; **o.** Colony on PDA from above and below. Scale bars: **c** = 200 μm, **d** = 100 μm, **e**–**h** = 20 μm, **i**–**n** = 10 μm.

Saprobic on dead frond stalks of *Alsophila costularis*. **Sexual morph**: Undetermined. **Asexual morph**: Hyphomycetous (Fig. 1), colonies on natural substrate superficial, effuse, gregarious, white. Conidiophores (268–)360–565 μm high (x̄ = 465 μm, n = 15), 9–14.5 μm wide (x̄ = 12 μm, n = 15) near the base, macronematous, unbranched, solitary, erect, straight or slightly flexuous, monophialidic, subcylindrical, thick-walled, 1–4-septate, dark brown to black, darker near the base, becoming paler brown towards the apex. Conidiogenous cells 25–54 × 7–11.5 μm (x̄ = 38 × 9.5 μm, n = 20), enteroblastic, monophialidic, terminal, swollen, with a shallow collarette, subcylindrical with apical taper to truncate apex, pale brown, rough. Conidia 20–24 × 10–12 μm (x̄ = 22 × 11.7 μm, n = 30), oblong to obovoid or ellipsoidal, occasionally with a median or submedian constriction, thick-walled, hyaline, aseptate, rough-walled.

Culture characteristics: Conidia germinating on PDA within 12 hours at 25℃, with hyaline germ tube germinating from the base of conidia. Colonies growing on PDA at 25℃, circular, flat surface, planar, thin, dark brown, reaching 2 cm diam. in 7 days, edge entire, emission at margin, dark brown to pale brown in reverse from the centre to margin of the colony.

Material: ex-type living culture, MFLUCC 21–0022.

#### Etymology

Referring to the host, which is a pteridophyte.

#### Notes

**Figure 2.**
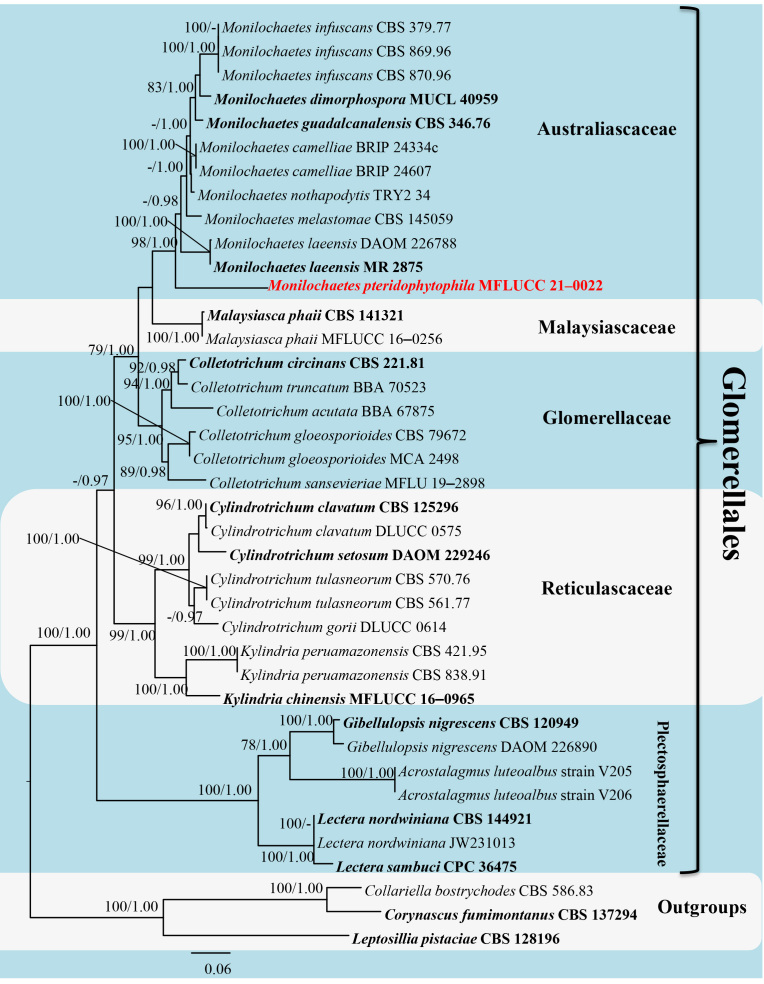
Phylogenetic tree generated from ML analysis, based on a concatenated LSU–ITS–SSU–RPB2 dataset. BS ≥ 70/PP ≥ 0.95 are indicated at the nodes. The newly-generated strain is shown in red and bold. Ex-type strains are indicated by black and bold. *Collariella bostrychodes* (CBS 586.83), *Corynascus fumimontanus* (CBS 137294) and *Leptosillia pistaciae* (CBS 128196) were used as outgroup taxa.

*Monilochaetes pteridophytophila* formed a distinct phylogenetic clade, which clustered with other species of *Monilochaetes* (Fig. 2). Following BLASTn searches, the closest matches of *M. pteridophytophila* are *M. melastomae* (LSU, NG_068601, 98.21% shared identity; ITS, NR_161124, 84.5%), *M. laeensis* (SSU, GU180610, 99.4%) and *M. infuscans* (RPB2, GU180658, 80.64%). *Monilochaetes pteridophytophila* is most similar to *M. regenerans* in the shape of conidiophores, conidiogenous cells and conidia (Bhat and Kendrick 1993). However, *M. pteridophytophila* has darker and longer conidiophores [(268–)360–565 μm vs. 300 μm high], shorter conidiogenous cells (25–54 μm vs. 70–100 μm) and smaller conidia (20–24 × 10–12 μm vs. 25–38 × 12–16 μm). Therefore, we introduce *M. pteridophytophila* as a new species, based on both phylogenetic and morphological evidence.

## Analysis

**Analysis Ⅰ: Phylogenetic reconstruction of a combined LSU, ITS, SSU and RPB2 sequence dataset**

The aligned, concatenated sequence matrix comprised sequence data for 39 taxa from seven families of the following loci: LSU (853 bp), ITS (489 bp), SSU (1,014 bp) and RPB2 (1,061 bp). Included sequences represented taxa of Glomerellales and three outgroup taxa, *Collariella bostrychodes* (CBS 586.83), *Corynascus fumimontanus* (CBS 137294) and *Leptosillia pistaciae* (CBS 128196). The sequence matrix comprised 3,417 characters (including gaps), of which 2,317 characters were constant, 185 variable characters were parsimony-uninformative and 915 characters were parsimony-informative. The matrix had 1,188 distinct alignment patterns, with 40.80% undetermined characters or gaps. The ML and BI analyses of the concatenated LSU–ITS–SSU–RPB2 dataset resulted in similar tree topologies (Fig. 2).

The phylogenetic tree shows that all strains of *Monilochaetes* clustered within Australiascaceae. The new species *M. pteridophytophila* forms a distinct clade, basal to other species of *Monilochaetes* with BS = 98% MLBS and PP = 1.00 (Fig. 2).

## Discussion

*Monilochaetes* is a widespread genus, with species occurring as endophytes, pathogens or saprobes on various plants in terrestrial environments (Rashmi et al. 2019, Table 3). All currently-described species of *Monilochaetes* have hyphomycetous asexual morphs. Only *M. camelliae*, *M. dimorphospora* and *M. nothapodytis* have dimorphic hyphomycetous asexual forms (Réblová et al. 2011a, Réblová et al. 2011b, Zhou et al. 2017). *Monilochaetes camelliae* and *M. laeensis* are represented also by sexual morphs (Sivanesan and Alcorn 2002, Réblová et al. 2011a).

*Monilochaetes pteridophytophila* is the second species found on a tree fern; *M. laeensis* occurs on tree ferns in Australia and the UK (Kirk 1986, Réblová et al. 2011a). *Monilochaetes pteridophytophila* forms a distinct clade with *M. laeensis*, basal to other *Monilochaetes* species. However, *M. pteridophytophila* differs from *M. laeensis* in having darker and longer conidiophores [(268–)360–565 μm vs. 40–160(–280) μm]. Hyde et al. (2018) and Hyde et al. (2020b) showed high fungal diversity in Thailand and suggested that studies on new hosts and new areas would lead to discovery of further new fungal species. Further studies of fungi on pteridophytes are likely expected to reveal more novel species.

Glomerellales was proposed by Réblová et al. (2011a) to accommodate three families, based on morphology and multilocus phylogenetic data: Australiascaceae, Glomerellaceae and Reticulascaceae. Later, Maharachchikumbura et al. (2016) accepted Plectosphaerellaceae in Glomerellales, based on the analysis of a combined LSU–SSU–TEF1–RPB2 dataset. Malaysiascaceae was added to Glomerellales by Tibpromma et al. (2018), based on a combined ribosomal DNA dataset (SSU, ITS, LSU). Our phylogenetic study confirms Glomerellales as a robust clade (ML = 100, PP = 1.00) comprising five lineages: Australiascaceae (ML = 98, PP = 1.00), Glomerellaceae (ML = 95, PP = 1.00), Malaysiascaceae (ML = 100, PP = 1.00), Plectosphaerellaceae (ML = 100, PP = 1.00) and Reticulascaceae (ML = 99, PP = 1.00). The phylogenetic relationships of families in Glomerellales are in agreement with Tibpromma et al. (2018) and Hyde et al. (2020a).

The tree topologies resulting from the phylogenetic reconstruction of a combined LSU–ITS dataset (analysis Ⅱ, Suppl. material 1) and the concatenated LSU–ITS–SSU–RPB2 dataset (analysis Ⅰ, Fig. 2) were overall similar and not significantly different. A comparison of phylogenetic analysis Ⅰ and Ⅱ with the analysis by Hyde et al. (2020a) showed negligible variation in tree topologies in Glomerellales, even with the inclusion of SSU and RPB2 data. The phylogeny in the current study suggests that LSU and ITS sequences can resolve interspecific relationships within *Monilochaetes*, as well as interfamilial relationships within Glomerellales.

## Supplementary Material

41E39E49-68F5-5173-9A62-ECE9231A3F0B 10.3897/BDJ.9.e67248.suppl1 Supplementary material 1 Phylogenetic analysis of a combined LSU and ITS sequence data Data type phylogenetic tree Brief description Analysis Ⅱ: Phylogenetic analysis of a combined LSU and ITS sequence data
The aligned sequence matrix comprises LSU (853 bp) and ITS (489 bp) sequence data for 39 taxa from GenBank. The aligned sequence matrix comprises 1,342 characters after alignment including the gaps, of which 873 characters were constant, 67 variable characters were parsimony-uninformative and 402 characters were parsimony informative. The matrix had 518 distinct alignment patterns, with 10.95% undetermined characters or gaps. The RAxML and BI analyses, based on combined LSU and ITS sequence data, provided similar tree topologies and the result of ML analysis is shown in FIGURE S1. File: oo_558189.docx https://binary.pensoft.net/file/558189 Jingyi Zhang

XML Treatment for Monilochaetes pteridophytophila

## Figures and Tables

**Table 1.** Primers and PCR amplification condition.

**Locus**	**Primers (forward/reverse)**	**PCR amplification condition**	**Reference(s)**
Large subunit ribosomal RNA (LSU)	LR0R/LR5	1. 95°C – 3 min	Vilgalys and Hester (1990), Hopple (1994), Lu et al. (2017)
		2. 94°C – 30 sec	
		3. 51°C – 50 sec	
		4. 72°C – 1 min	
		5. Repeat 2–4 for 30 cycles	
		6. 72°C – 7 min	
		7. 4°C on hold	
Internal transcribed spacer region of ribosomal DNA (ITS)	ITS1/ITS4	1. 95°C – 3 min	White et al. (1990), Lu et al. (2017)
		2. 95°C – 30 sec	
		3. 51°C – 1 min	
		4. 72°C – 45 sec	
		5. Repeat 2–4 for 34 cycles	
		6. 72°C – 10 min	
		7. 4°C on hold	
Small subunit ribosomal RNA (SSU)	NS1/NS4	1. 94°C – 3 min	White et al. (1990)
		2. 94°C – 45 sec	
		3. 56°C – 50 sec	
		4. 72°C – 1 min	
		5. Repeat 2–4 for 40 cycles	
		6. 72°C – 10 min	
		7. 4°C on hold	
RNA polymerase II second largest subunit (RPB2)	fRPB2-5f/fRPB2-7cR	1. 95°C – 5 min	Liu et al. (1999)
		2. 95°C – 1 min	
		3. 55°C – 2 min	
		4. 72°C – 90 sec	
		5. Repeat 2 – 4 for 40 cycles	
		6. 72°C – 10 min	
		7. 4°C on hold	

**Table 2.** Taxa used to infer the phylogenetic tree and their GenBank accession numbers. **Notes**: "-" as meaning no data available in GenBank. The newly-generated sequences are underlined. The ex-type strains are in bold.

**Taxa**	**Strain/ Voucher No.**	**GenBank Accession no.**			
		**ITS**	**LSU**	**SSU**	**RPB2**
*Acrostalagmus luteoalbus*	strain V205	KJ443271	KJ443141	KJ443096	KJ443184
*Acrostalagmus luteoalbus*	strain V206	KJ443272	KJ443142	KJ443097	KJ443185
*Collariella bostrychodes*	CBS 586.83	KX976642	KX976739	-	KX976838
*Colletotrichum acutatum*	BBA 67875	AJ301926	AJ301926	AJ301926	-
***Colletotrichum circinans***	**CBS 221.81**	**NR_111457**	**NG_069094**	**NG_062845**	-
*Colletotrichum gloeosporioides*	CBS 79672	-	AY705727	-	-
*Colletotrichum gloeosporioides*	MCA 2498	DQ286198	DQ286199	-	-
*Colletotrichum sansevieriae*	MFLU 19–2898	MT177931	MT177958	MT177985	MT432208
*Colletotrichum truncatum*	BBA 70523	AJ301937	AJ301937	AJ301937	-
***Corynascus fumimontanus***	**CBS 137294**	**MK919291**	**LK932706**	-	**MK919347**
***Cylindrotrichum clavatum***	**CBS 125296**	**GU180627**	**GU180643**	**GU180622**	-
*Cylindrotrichum clavatum*	DLUCC 0575	MH120193	MH120184	-	MH120179
*Cylindrotrichum gorii*	DLUCC 0614	MH120195	MH120189	-	MH120183
*Cylindrotrichum oligospermum*	CBS 570.76	MH861002	MH872775	-	-
*Cylindrotrichum oligospermum*	CBS 561.77	GU291801	-	-	-
***Cylindrotrichum setosum***	**DAOM 229246**	-	**GU180652**	**GU180617**	-
***Gibellulopsis nigrescens***	**CBS 120949**	**NR_149327**	**NG_067330**	-	**LR026149**
*Gibellulopsis nigrescens*	DAOM 226890	GU180631	GU180648	GU180613	GU180664
***Kylindria chinensis***	**MFLUCC 16–0965**	**MH120190**	**MH120186**	-	**MH120181**
*Kylindria peruamazonensis*	CBS 838.91	GU180628	GU180638	GU180609	GU180656
*Kylindria peruamazonensis*	CBS 421.95	GU291800	HM237325	-	-
***Lectera nordwiniana***	**CBS 144921**	**NR_161150**	**NG_066300**	-	**MK047549**
*Lectera nordwiniana*	JW231013	MK047462	MK047512	-	MK047550
***Lectera sambuci***	**CPC 36475**	**NR 170055**	**MT223905**	-	-
***Leptosillia pistaciae***	**CBS 128196**	**NR 160064**	**MH798901**	-	**MH791334**
***Malaysiasca phaii***	**CBS 141321**	**KX228280**	**KX228331**	-	-
*Malaysiasca phaii*	MFLUCC 16–0256	MH275069	MH260302	MH260342	-
*Monilochaetes camelliae*	BRIP 24607	HM237327	HM237324	-	-
*Monilochaetes camelliae*	BRIP 24334c	HM237326	HM237323	-	-
***Monilochaetes dimorphospora***	**MUCL 40959**	**NR_137765**	**HQ609480**	**NG 062390**	-
***Monilochaetes guadalcanalensis***	**CBS 346.76**	**GU180625**	**GU180640**	-	-
*Monilochaetes infuscans*	CBS 379.77	-	GU180645	GU180619	GU180658
***Monilochaetes infuscans***	**CBS 870.96**	-	**GU180644**	**GU180621**	-
*Monilochaetes infuscans*	CBS 869.96	GU180626	GU180639	GU180620	GU180657
***Monilochaetes laeensis***	**MR 2875**	**GU180624**	**GU180642**	-	-
*Monilochaetes laeensis*	DAOM 226788	GU180623	GU180641	GU180610	-
***Monilochaetes melastomae***	**CBS 145059**	**NR_161124**	**NG_068601**	-	-
***Monilochaetes nothapodytis***	**TRY2 34**	**MF153475**	**MF153476**	-	-
***Monilochaetes pteridophytophila***	**MFLUCC 21 – 0022**	**MW826218**	**MW826219**	**MW826220**	**MW829186**

**Table 3.** Synopsis of asexual morph of accepted species in *Monilochaetes* with morphological features.

**Species**	**Hosts**	**Distribution**	**Macroconidiophores/ Microconidiophores (μm)**	**Macroconidia/ Microconidia (μm)**	**Reference(s)**
*Monilochaetes basicurvata*	Palm petiole	Peru	200–300(–600) × 5–7 / -	9–25 × 3.5–6(–7) / -	Matsushima (1995)
*M. camelliae*	Branch of *Camellia sinensis*	Australia	200–720 × 9–10(–10.5) / 40–60 × 2–2.5	20.5–24(–26.5) × (10–)11–12 / 4–5.5 × 3–3.5	Sivanesan and Alcorn (2002), Réblová et al. (2011a)
*M. dimorphospora*	Decayed wood	Cuba	230–450 × 6.5–7 / 40 × 3	21–25(–27) × 6.5–7 / 4.5–6(–6.5) × 2.5–3	Réblová et al. (2011b)
*M. guadalcanalensis*	Decaying leaf of *Musa* sp.	Solomon Islands	150–220(–400) × 4–7 / -	18–21 × 6–9 /-	Rong and Gams (2000)
*M. infuscans*	*Ipomoea batatas* (sweet potato)	Asia, Australia, Europe, New Zealand, South Africa, Pacific Islands, USA	60–400 / -	15–20 × 4–6 / -	Harter (1916), Lawrence et al. (1981), Rong and Gams (2000)
*M. laeensis*	Leaf litter, dead stipes and spathes of a tree fern, rotting frond stems of *Victoria regia*, dead stipes of *Dicksonia antarctica* and dead palm spathes	Australia, British Isles, Cuba, Ethiopia, India, Malaysia, Papua New Guinea, Sabah and Sri Lanka.	40–160(–280) × 7–8 / -	(15.5–)18–22.5(–23.5) × 7.5–9(–10) / -	Bhat and Sutton (1985), Kirk (1986), Rong and Gams (2000), Réblová et al. (2011a)
*M. melastomae*	Leaf spots of *Melastoma* sp.	Malaysia	90 – 250 × 6 –10 /-	(17–)18–19(–20) × (7.5–)8 / -	Crous et al. (2018)
*M. nothapodytis*	Healthy leaf of *Nothapodytes pittosporoides*	China	300–640 × 7.5–13 / 18–35 × 4–5.5	16.5–24 × 9.5–15.5 / 3–4.9 × 2.9–4	Zhou et al. (2017)
***M. pteridophytophila***	**Dead frond stalks of *Alsophila costularis***	**Thailand**	**(268–)360–565 × 9–14.5** / -	**20–24 × 10–12** / -	**This study**
*M. regenerans*	Dead twigs of *Ficus* sp.	India	300 × 8–10 / -	25–38 × 12–16 / -	Bhat and Kendrick (1993)
