# Supplementary material for: Monilochaetes pteridophytophila (Australiascaceae, Glomerellales), a new fungus from tree fern
Source: Biodivers Data J. 2021 Jul 30;9:e67248. doi: 10.3897/BDJ.9.e67248 (PMC8346447; doi:10.3897/BDJ.9.e67248)
Supplement: Supplementary material 1 — Phylogenetic analysis of a combined LSU and ITS sequence data [file bdj-09-e67248-s001.docx]

***Monilochaetes pteridophytophila* (Australiascaceae, Glomerellales), a new fungus from tree fern**

Jingyi Zhang, Rungtiwa Phookamsak, Ausana Mapook, Yongzhong Lu & Menglan Lv

**Supplementary:**

**
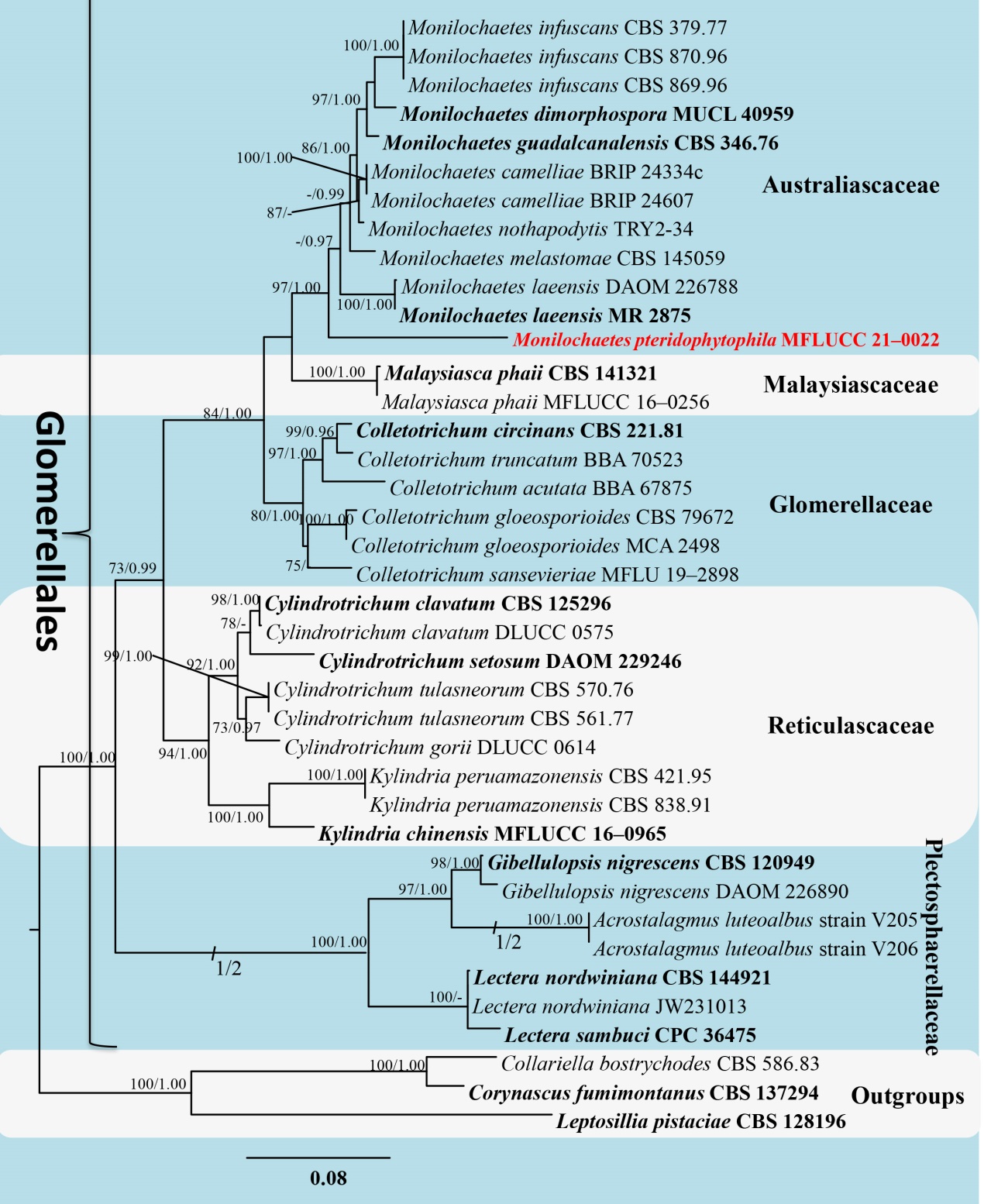
**

**FIGURE S1.** Phylogenetic tree generated from maximum likelihood (ML) analysis based on a concatenated LSU-ITS-SSU-RPB2 sequences dataset. Bootstrap values for maximum likelihood equal to or greater than 70% and Bayesian posterior probability equal to or greater than 0.95 PP are indicated at the nodes as MLBS/PP. Hyphen (“-”) indicates a value lower than 70% for MLBS and a posterior probability lower than 0.95 PP for BI analyses. The newly generated strain is shown in red bold. Ex-type strains are indicated by black bold. *Collariella bostrychodes* (CBS 586.83), *Corynascus fumimontanus* (CBS 137294) and *Leptosillia pistaciae* (CBS 128196) are used as outgroup taxa
